# Assessment of the Potential of Vaccination to Combat Antibiotic Resistance in Gonorrhea: A Modeling Analysis to Determine Preferred Product Characteristics

**DOI:** 10.1093/cid/ciz1241

**Published:** 2020-01-06

**Authors:** Lilith K Whittles, Peter J White, Xavier Didelot

**Affiliations:** 1 Department of Infectious Disease Epidemiology, School of Public Health, Imperial College London, London, United Kingdom; 2 Medical Research Council Centre for Global Infectious Disease Analysis, School of Public Health, Imperial College London, London, United Kingdom; 3 National Institute for Health Research Health Protection Research Unit in Modelling Methodology, School of Public Health, Imperial College London, London, United Kingdom; 4 Modelling and Economics Unit, National Infection Service, Public Health England, London, United Kingdom; 5 School of Life Sciences, University of Warwick, Coventry, United Kingdom; 6 Department of Statistics, University of Warwick, Coventry, United Kingdom

**Keywords:** gonorrhea, vaccination, antibiotic resistance, transmission model, treatment failure

## Abstract

**Background:**

Gonorrhea incidence is increasing rapidly in many countries, while antibiotic resistance is making treatment more difficult. Combined with evidence that two meningococcal vaccines are likely partially protective against gonorrhea, this has renewed interest in a gonococcal vaccine, and several candidates are in development. Key questions are how protective and long-lasting a vaccine needs to be, and how to target it. We assessed vaccination’s potential impact and the feasibility of achieving the World Health Organization’s (WHO) target of reducing gonorrhea incidence by 90% during 2018–2030, by comparing realistic vaccination strategies under a range of scenarios of vaccine efficacy and duration of protection, and emergence of extensively-resistant gonorrhea.

**Methods:**

We developed a stochastic transmission-dynamic model, incorporating asymptomatic and symptomatic infection and heterogeneous sexual behavior in men who have sex with men (MSM). We used data from England, which has a comprehensive, consistent nationwide surveillance system. Using particle Markov chain Monte Carlo methods, we fitted to gonorrhea incidence in 2008–2017, then used Bayesian forecasting to examine an extensive range of scenarios.

**Results:**

Even in the worst-case scenario of untreatable infection emerging, the WHO target is achievable if all MSM attending sexual health clinics receive a vaccine offering ≥ 52% protection for ≥ 6 years. A vaccine conferring 31% protection (as estimated for MeNZB) for 2–4 years could reduce incidence in 2030 by 45% in the worst-case scenario, and by 75% if > 70% of resistant gonorrhea remains treatable.

**Conclusions:**

Even a partially-protective vaccine, delivered through a realistic targeting strategy, could substantially reduce gonorrhea incidence, despite antibiotic resistance.

## Teaser

A gonorrhea vaccine offering partial protection for several years, pragmatically targeted to people testing for gonorrhea due to symptoms or screening due to sexual risk behavior, could substantially reduce gonorrhea incidence, combating increases in antibiotic resistance.

The World Health Organization (WHO) classifies *Neisseria gonorrhoeae* as a priority bacterial pathogen, due to the high global burden of infection combined with evolution and global spread of resistance to every antibiotic historically used against it [1, 2]. Countries such as the United States [3], Australia [4], the United Kingdom (UK) [5], and other European countries [6] have reported rapidly growing epidemics, particularly among men who have sex with men (MSM).

Due to the threat of antibiotic-resistant (ABR) gonorrhea, it has been suggested that vaccination may be the only sustainable solution to gonorrhea control [7]. Vaccine development has been hampered by genetic variability in the gonococcus and the lack of a measurable correlate of protection and a suitable animal model [8]. None of 4 vaccine candidates that progressed to clinical trials was effective [9]. However, indication that vaccination against gonorrhea might be feasible came from surveillance reports from Cuba and New Zealand showing a decline in gonorrhea incidence following vaccination initiatives against the closely related *Neisseria meningitidis* [10–12]. A retrospective case-control study of 15 000 young adults in New Zealand who had received an outer-membrane vesicle meningococcal B vaccine (MeNZB) estimated 31% (95% confidence interval [CI], 21%–39%) protection against *N. gonorrhoeae* [13], and a recent study reported that the Bexsero meningococcal B vaccine may be more protective than this [14]. There are now multiple vaccine candidates in preclinical development [11, 15].

Key questions to inform development and use of vaccines to control gonorrhea are the preferred product characteristics of a gonorrhea vaccine (ie, what efficacy and duration of protection are required) [16] and how best to deploy vaccines to decrease the overall burden of disease [17–19]. In 2016, the WHO announced a global health sector strategy on sexually transmitted infections, with a target of 90% reduction in gonorrhea incidence by 2030 [2]. We investigated how protective and long-lasting a vaccine would need to be to reduce total incidence below the WHO target using a stochastic model (accounting for variability due to random chance) of gonorrhea epidemiology in MSM in England calibrated to surveillance data [20, 21], with varying future levels of antibiotic resistance. We compared the impact and efficiency of 3 realistic vaccination strategies, studied the interplay between vaccination and antibiotic resistance levels, and quantified the effect of differing levels of vaccine uptake. Finally, we assessed the potential impact of vaccines with a partially protective profile similar to MeNZB.

## METHODS

### Model Structure

We developed a stochastic compartmental transmission-dynamic model to project the future course of a gonorrhea epidemic under different vaccination scenarios, considering antibiotic-sensitive and ABR strains. We used data from England, which has a comprehensive, consistent, nationwide surveillance system [20, 21]. We considered transmission within MSM as they have the highest per-capita rate of infection. We simulated gonorrhea transmission from 2008 to 2030, using surveillance data from the period 2008–2017 for calibration and then projecting scenarios to 2030. We extended a previous model [22] to incorporate heterogeneity in sexual risk behavior by dividing the population into low- and high-risk groups with characteristic rates of sexual partner change, based on the Natsal-3 survey [23].

**Figure 1.**
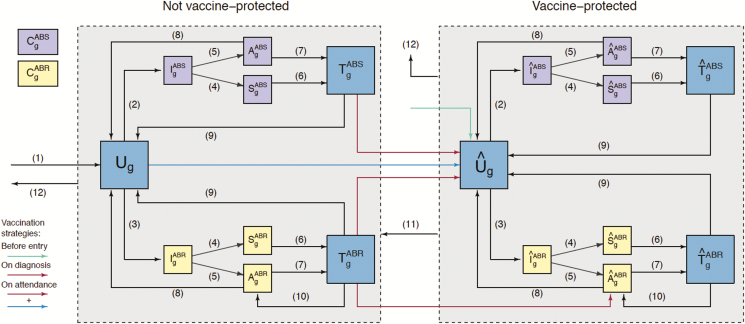
Model structure flow diagram. The population is divided into compartments representing different states, with changes of state occurring due to various processes. Individuals enter the sexually active population (arrow 1) at age 15 years. They are initially uninfected (*U*), and belong to a sexual activity group *g* (low or high). Individuals become infected with either the antibiotic-sensitive (ABS) strain (2) or antibiotic-resistant (ABR) strain (3). Infected individuals pass through an incubating state (*I*), before either developing symptoms (4) and entering the symptomatic infection state (*S*), or remaining asymptomatic (5) and entering the asymptomatic infection state (*A*). Symptomatic individuals seek treatment (6) and enter the treatment state (*T*). Asymptomatic infections can be identified through screening and treatment (7), with individuals entering the treatment state (*T*), or there can be natural recovery (8), returning individuals to the uninfected state (*U*). All treated infections are cured (9), with the exception of a proportion of ABR infections for which treatment fails, resulting in persistent infection (10). Depending on the vaccination strategy, individuals are vaccinated before entry into the sexually active population (in which case all vaccinees are uninfected), upon gonorrhea diagnosis (with vaccination given to those who are treated), or upon clinic attendance for gonorrhea screening (with all individuals attending clinics being eligible). Upon vaccination, individuals enter corresponding compartments indicated with a circumflex (^). Vaccine protection eventually wanes (11), with individuals moving into the corresponding compartments (without a circumflex). Individuals leave the sexually active population at age 65 years, regardless of infection or vaccination status (12).

Following acquisition of gonorrhea, individuals initially pass through a short incubation period, after which they either develop symptoms or remain asymptomatically infected (Figure 1). Surveillance data are not stratified by infection site (rectum, pharynx, urethra); hence, estimated parameters can be interpreted as an average across infection sites. Infected individuals are treated after seeking care due to symptoms or after testing positive in sexual health screening. Treated individuals become uninfected, except for a proportion of those infected with the ABR strain for whom treatment fails, leading to persistent infection for which the same dynamics are assumed as for asymptomatic cases [22]. Recovery from untreated infection also occurs naturally over time. Infection does not confer natural immunity [24, 25]. We consider a situation in which the ABR strain emerges globally in 2020 and is imported into the highly sexually active group.

### Model Calibration and Accounting for Uncertainty

We calibrated the model, in a Bayesian framework, to the annual number of gonorrhea cases in MSM in England between 2008 and 2017 [20]. These data were considered as the observed realizations of a complex underlying unobserved Markov process (Figure 1). Prior parameter distributions were based on published evidence where available, and uninformative priors used otherwise. All unknown parameters were calibrated, with a particle filter [26] used to produce an unbiased estimate of the likelihood of the observed data given the model, in a particle Markov chain Monte Carlo process, which produced a posterior sample of the model parameters given the observed data [27, 28]. We accounted for uncertainty in estimated parameters by using 1000 samples from the joint posterior distribution. Full details of the model, the parameter values, and prior distributions are shown in the Supplementary Materials. We varied the frequency of treatment failure for the ABR strain (0%–100%).

### Vaccination Scenarios

We considered 500 hypothetical vaccine profiles of varying protection (1%–100%) and duration (1–20 years). Partial protection was assumed to be “leaky” (ie, degree-type) [29], with all vaccinees being less likely, but still able, to acquire infection. Vaccine protection only affects the probability of acquisition and does not affect progression through stages of infection after acquisition.

We considered 3 realistic strategies for the vaccine deployment: “vaccination before entry” into the sexually active population (where adolescents are vaccinated before they become sexually active); “vaccination on diagnosis” with gonorrhea (a practical strategy designed to target those most at risk, as gonorrhea diagnosis is used as a proxy measure for risk of future exposure); and “vaccination on attendance” at a sexual health clinic for any reason, including seeking testing and treatment for symptoms or asymptomatic screening (which broadens the eligibility criteria of the “vaccination on diagnosis” strategy to include all clinic attendees). Vaccine uptake among eligible individuals was varied (50%–100%) for each strategy. Scenarios were compared to baseline projections without vaccination.

## RESULTS

**Figure 2.**
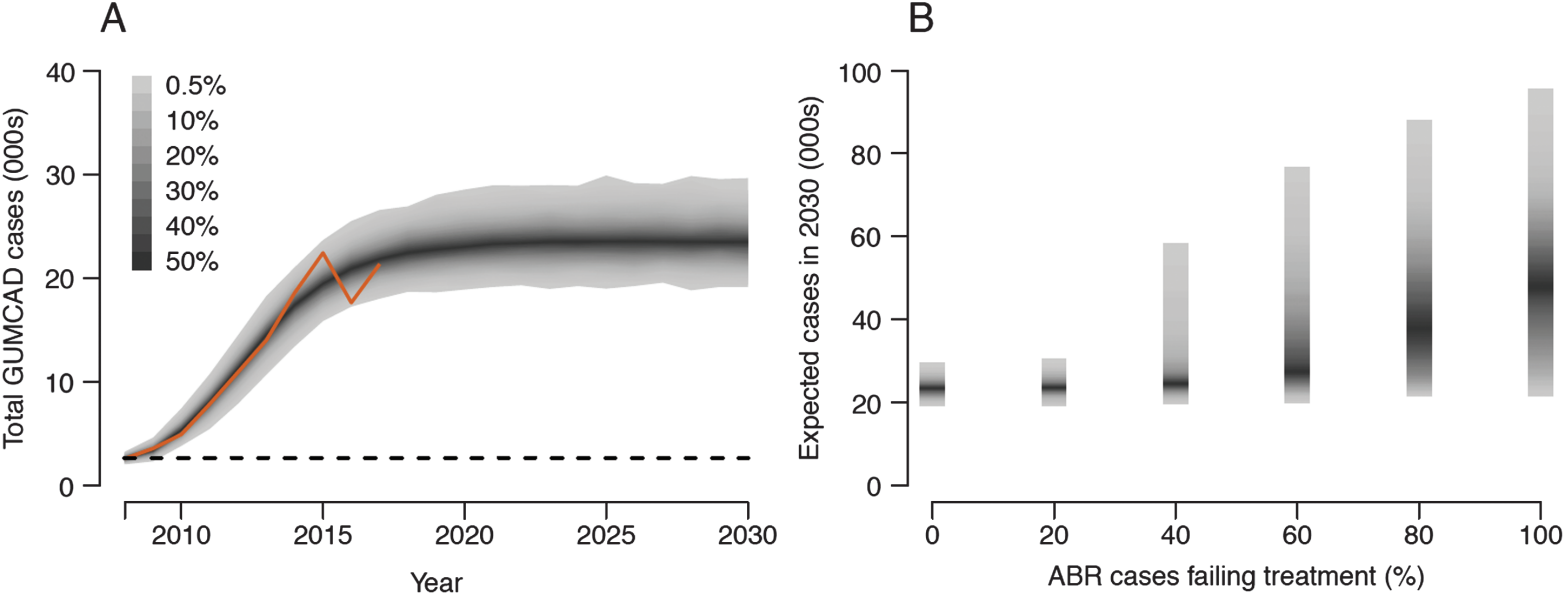
Simulated annual gonorrhea cases between 2008 and 2030 in the absence of vaccination. The model is fitted to data in the period 2008–2017 and then projected beyond that period. *A*, Number of cases if a novel resistant strain does not emerge. The solid line depicts surveillance data; the horizontal line shows the World Health Organization target of 90% reduction in incidence relative to 2018. Shaded areas show 99% posterior predictive intervals, based on 1000 simulations. *B*, Expected number of cases in 2030 depending on the frequency of treatment failure for a novel antibiotic-resistant strain emerging in 2020. Abbreviations: ABR, antibiotic-resistant; GUMCAD, the Genitourinary Medicine Clinical Activity Dataset.

The model was successfully calibrated, with simulated epidemic curves being in close agreement with the observed data for all years (Figure 2A). For example, the model estimated 21 900 (95% CI, 18 900–25 200) cases in 2017, in good agreement with the data (21 300 cases) [20].

In the best-case scenario, with no worsening of resistance, the model predicted 23 500 (20 000–27 600) cases in 2030. However, emergence of an ABR strain causing treatment failures would increase the projected number of cases (Figure 2B): The higher the frequency of treatment failure, the greater the onward transmission of drug-resistant infections and the greater the predicted number of cases. In the worst-case scenario of 100% treatment failure for the ABR strain, the model predicted 48 500 (23 600–80 100) gonorrhea cases in 2030.

**Figure 3.**
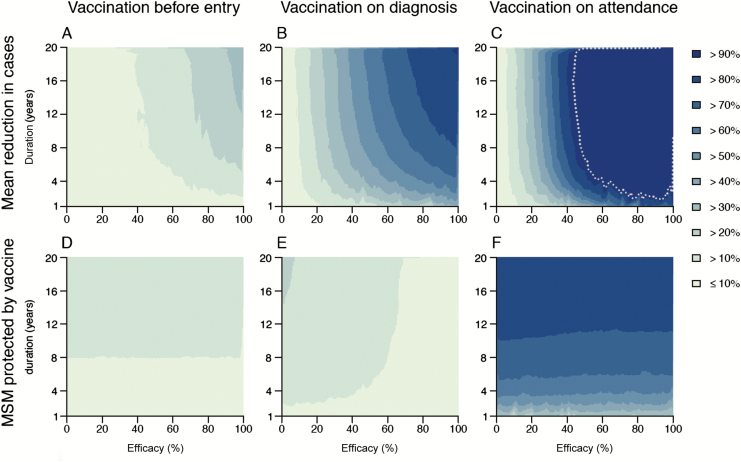
Impact of different vaccination strategies against gonorrhea, given the emergence of a resistant strain in 2020 for which antibiotic treatment always fails. Vaccination was implemented from 2020 and administered to individuals entering the sexually active population (*A* and *D*), or to patients diagnosed with gonorrhea (*B* and *E*), or to patients on clinic attendance (*C* and *F*). Level (horizontal axes) and duration of protection (vertical axes) were varied. *A–C*, Mean reduction in the expected number of cases in 2030. *D–F*, Proportion of men who have sex with men protected by the vaccine in 2030. Vaccine profiles (ie, combinations of level and duration of protection) for which the World Health Organization incidence target was achieved by 2030 are highlighted with a dotted line (this is only achieved in part of *C*). Abbreviation: MSM, men who have sex with men.

Focusing on the worst-case scenario where treatment of ABR gonorrhea always failed, we assessed the impact of each hypothetical vaccine profile (ie, combination of level and duration of protection) under the 3 deployment strategies by calculating the expected reduction in gonorrhea cases in 2030 (Figure 3) compared with no vaccination. Achieving the WHO target corresponds to < 2600 cases in MSM in England in 2030 [2]. In the vaccination-before-entry strategy, even a fully protective vaccine lasting 20 years achieved only a 34% (17%–52%) reduction in expected cases in 2030 (Figure 3A), well below the WHO target. Under this strategy, only adolescents entering the sexually active population are vaccinated, so vaccine coverage across the entire MSM population is slow to accumulate, reducing the impact of the intervention. Vaccination on diagnosis with this idealized vaccine also fell short of the WHO target but achieved a much higher reduction of 92% (82%–98%) (Figure 3B). By extending vaccination to all MSM tested for gonorrhea (ie, vaccination on attendance, both those seeking care for symptoms and those screened in the absence of symptoms), the WHO target could be achieved using a ≥ 52% effective vaccine protecting for ≥ 6 years, or equivalently a ≥ 70% effective vaccine lasting ≥ 3 years (Figure 3C). Under the vaccination-on-diagnosis and vaccination-on-attendance strategies, a vaccine lasting 8 years had similar benefits to one offering the same protection for a longer period (Figure 3B and 3C).

Under the vaccination-before-entry strategy, the proportion of MSM protected in 2030 was < 20% irrespective of the duration of vaccine protection (Figure 3D), because most individuals were already sexually active and therefore ineligible for vaccination. Under the vaccination-on-diagnosis strategy, the protected proportion was generally higher (Figure 3E) because more individuals were eligible for vaccination. Interestingly, the proportion protected decreased as vaccine efficacy increased, because a highly protective vaccine reduced transmission, which in turn reduced the number of cases treated and hence individuals vaccinated alongside treatment. This effect also occurred under the vaccination-on-attendance strategy, but it was much less pronounced as patients attending for screening were also vaccinated. The maximum proportion protected under vaccination on attendance was 86% (85.8%–86.4%), if the duration of protection were 20 years (Figure 3F).

**Figure 4.**
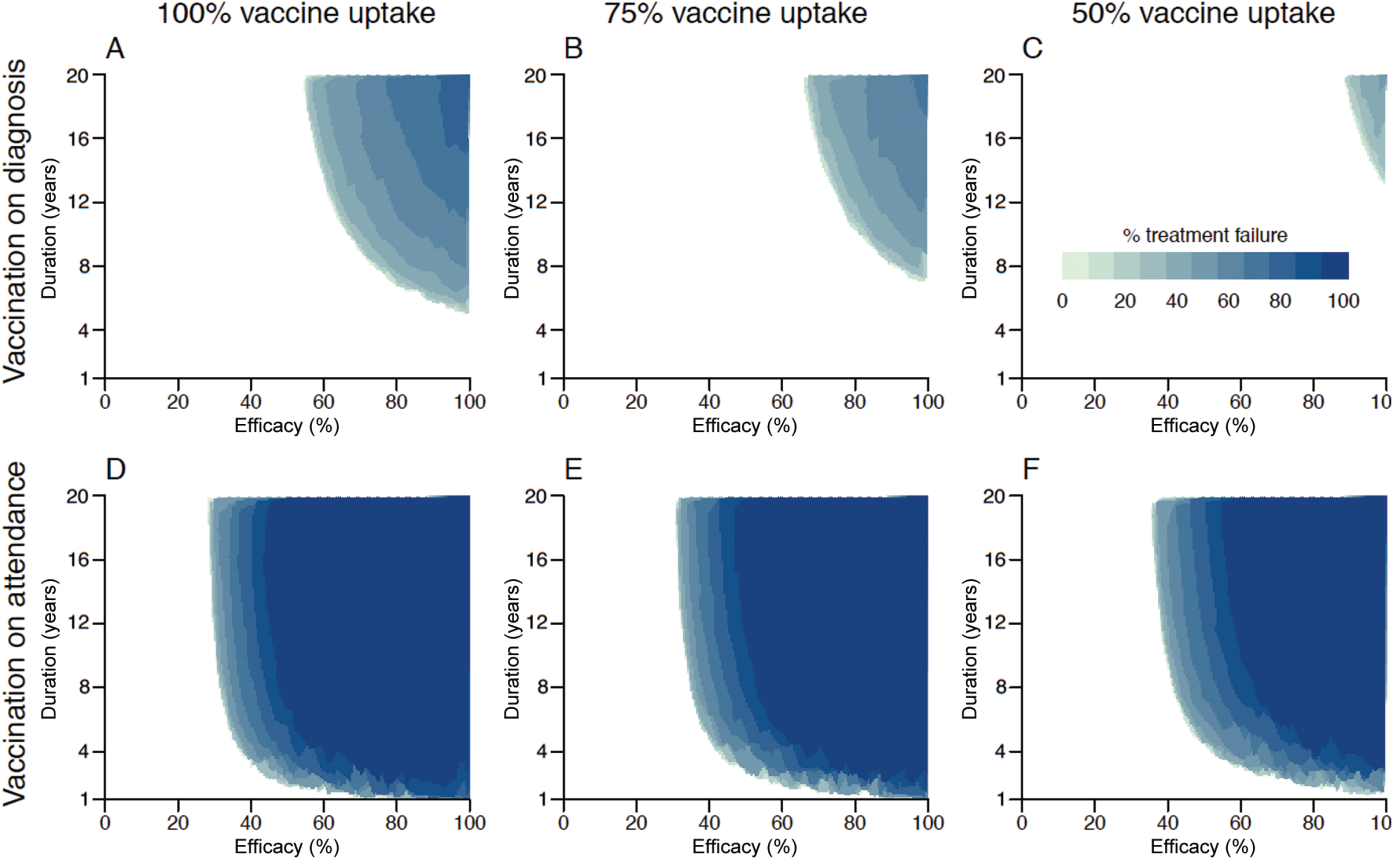
Protection level and duration of vaccine needed to reduce incidence below the World Health Organization (WHO) target by 2030 for gonorrhea strains with varying degrees of resistance. Results are shown for vaccination on diagnosis (*A–C*) or on clinic attendance (*D–F*), under varying levels of vaccine uptake: 100% (*A* and *D*), 75% (*B* and *E*), and 50% (*C* and *F*). The vaccination-before-entry strategy is not shown as it did not achieve the WHO target for any scenario considered.

We assessed how the impact of vaccination would differ depending on the treatability of the emergent gonorrhea ABR strain and the uptake of vaccination by eligible MSM (Figure 4). The less treatable the ABR strain, the more protective a vaccine would need to be to meet the WHO target. The vaccination-before-entry strategy was unable to achieve the WHO target, no matter how protective and long-lasting the vaccine, even in the best-case scenario of no treatment failure. The vaccination-on-diagnosis strategy met the WHO target for a range of vaccine profiles, in scenarios where at least 20% of ABR cases were treatable, provided all eligible MSM accepted the vaccine when offered and protection lasted almost 20 years (Figure 4A). With 75% vaccine uptake, vaccination on diagnosis could not meet the target if ABR treatment failure exceeded 70% (Figure 4B). With an uptake of only 50%, the target could only be met provided treatment failure remained below 50% (Figure 4C). The lower the uptake, the greater the vaccine protection level and/or duration required to achieve a given impact (Figure 4A–C). The vaccination-on-attendance strategy resulted in much higher coverage, meaning that less-protective vaccines were able to achieve the WHO target (Figure 4D–F). For example, in the absence of treatment failure, a 31% protective vaccine lasting 10 years could meet the WHO target (Figure 4D), whereas under the vaccination-on-diagnosis strategy, the vaccine would need to be 67% protective (Figure 4A). At the other extreme, if treatment of ABR cases always failed, then the WHO target could be achieved with the vaccination-on-attendance strategy but would require, for example, a protection duration of 6 years and efficacy of 50%–70% depending on uptake (Figure 4D–F).

**Figure 5.**
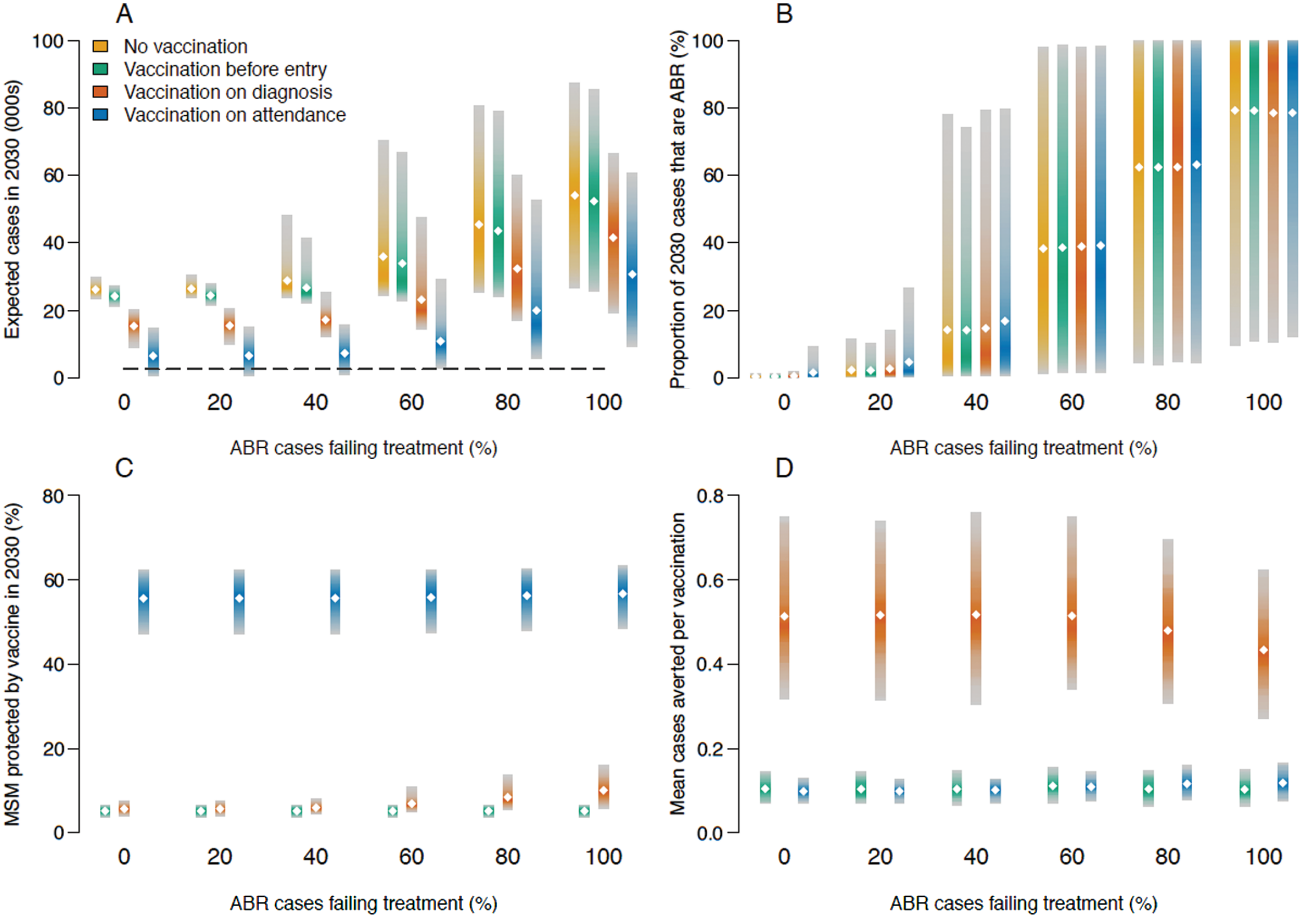
Potential impact and efficiency of an outer-membrane vesicle meningococcal B (MeNZB)–like vaccine. Vaccination was implemented before entry into the sexually active population, on diagnosis, or on clinic attendance. Shaded bars depict the 95% predictive interval; diamonds indicate the mean. *A*, Expected gonorrhea cases in 2030, with the dashed line depicting the World Health Organization target. *B*, Proportion of gonorrhea cases expected to be resistant in 2030. *C*, Proportion of men who have sex with men protected by the vaccine in 2030. *D*, Mean cases averted per course of vaccination. Abbreviations: ABR, antibiotic-resistant; MSM men who have sex with men.

Currently, the only vaccine with estimated effectiveness of protection against gonorrhea is MeNZB, which offers 21%–39% protection [13]. While there are no robust estimates of the duration of protection of MeNZB, we modeled a conservative 2- to 4-year duration in line with initial indications [13]. We considered deployment of a similar vaccine using the 3 strategies and compared the expected number of cases in 2030, the proportion of antibiotic resistance, the proportion of protected MSM, and the number of cases averted over 10 years per person vaccinated (Figure 5). If there were no emergence of ABR gonorrhea, then a vaccine with properties like MeNZB could have a substantial impact on the projected epidemic. With uptake of 100%, vaccination on diagnosis would reduce the expected incidence in 2030 by 41% (18%–65%; from 23 500 [20 000–27 600] to 13 900 [8200–18 700]), and vaccination on attendance would reduce incidence by 75% (40%–98%) to 5900 (450–13 600) cases. The greater impact of vaccination on attendance was due to much greater coverage, with much lower efficiency: The mean number of cases averted per vaccination was 0.10 (0.07–0.13) compared with 0.51 (0.32–0.75) for vaccination on diagnosis. It should be noted that even vaccination on attendance was insufficient to meet the WHO target in 75% of the simulations. The vaccination-before-entry strategy was ineffective with an MeNZB-like vaccine, achieving only a 7% (0%–23%) reduction in 2030 incidence, even without emergence of ABR.

The greater the frequency of treatment failure for ABR infections, the greater the predicted incidence in 2030 and the less likely the epidemic could be controlled by a MeNZB-like vaccine (Figure 5A). Vaccination on attendance was always the most effective strategy, regardless of the treatability of the resistant strain. In the extreme case where ABR treatment always failed, vaccination on attendance with an MeNZB-like vaccine with 100% uptake reduced the expected number of cases by 45% (18%–77%). This equated to the prevention of around 20 900 cases in 2030, reducing the expected diagnoses from 48 500 (23 600–80 100) to 27 600 (8000–55 800). The proportion of cases expected to be ABR in 2030 was unaffected by vaccination, regardless of the vaccination strategy: Vaccination reduced the expected diagnoses of both the resistant and susceptible strains proportionately (Figure 5B). The proportion of individuals protected by the vaccine, and the corresponding number of vaccine doses dispensed, depended more on the vaccination strategy than on the treatability of the ABR strain (Figure 5C), with vaccination on attendance resulting in a much higher proportion of protected individuals. Vaccination on diagnosis proved to be the most efficient strategy overall, with on average about 1 averted case for every 2 vaccine doses administered, which remained constant for almost all resistant strains (Figure 5D).

## DISCUSSION

We developed a stochastic transmission-dynamic model of gonorrhea that incorporates heterogeneity in sexual behavior and considers use of health services for care-seeking and screening. The model was calibrated using 10 years’ data using Bayesian inference methods to estimate model parameters, and then used to examine the population-level impact of potential vaccines, administered via 3 realistic strategies. Our results show that even a partially protective vaccine could be valuable in controlling the gonorrhea epidemic and combating the spread of antibiotic resistance. Provided infections remain ultimately treatable, as vaccinating all MSM attending sexual health clinics from 2020 onward with a 40% protective vaccine lasting at least 4 years, or a 50% protective vaccine lasting at least 2 years, would be sufficient to meet the WHO target of a 90% reduction in annual incidence between 2018 and 2030 [2]. While this requires greater protection than the 31% (95% CI, 21%–39%) that MeNZB appears to offer [13], Bexsero is expected to be more protective due to its neisserial heparin binding antigen component [14]. In addition to protecting against infection with gonorrhea, vaccination with MeNZB might also be protective against severe disease if infection is nevertheless acquired [30].

We investigated the interplay between antibiotic resistance and vaccination, making the conservative (and likely pessimistic) assumption that antibiotic resistance did not incur a fitness cost. In the extreme case where treatment for the ABR strain always fails, a 52% protective vaccine lasting 6 years, administered to all MSM attending sexual health clinics, would be necessary to achieve the WHO target.

Imperfect vaccines are usually modeled as providing either take-type protection (where a proportion of vaccinees are fully protected and the remainder not at all), or degree-type protection (where all vaccinees have a partial reduction in the probability of infection upon exposure) [29]. Here we used the latter, even though it is unknown what type of protection a future gonorrhea vaccine might provide, or indeed if it would conform to one type or another or a mixture of both. For vaccines with degree-type protection, greater coverage is necessary to reduce the prevalence of infection than with take-type protection [31], so that our choice is conservative. Furthermore, a take-type protection vaccine is similar in result to the effect of partial uptake for a highly protective degree-type vaccine, which we considered separately (Figure 4). Degree-type protection could conceivably manifest as a reduction in infectiousness [32], rather than the reduction in susceptibility we model here; previous modeling has found the impact to be similar [17].

Uptake of vaccination is clearly a critical determinant for the success of any vaccination program, and societal concerns about vaccines are a potential barrier to achieving high coverage [12]. However, a recent pilot program for human papillomavirus vaccine in UK MSM recorded uptake of 45%, which is likely to be an underestimate due to incomplete recording [33]. Therefore, the 50% uptake scenario we modeled could be considered a realistic lower bound, especially for the vaccination-on-diagnosis and vaccination-on-attendance strategies, in which the vaccine would be offered to individuals likely to be concerned about gonorrhea infection.

Our study is the first to consider vaccination against gonorrhea in the context of antibiotic resistance, and to assess the potential real-world impact that could be achieved using a vaccine with protection profile similar to MeNZB as well as potentially superior gonorrhea vaccines, which are expected to include Bexsero [14]. Previous modeling studies have focused on assessing the potential impact of hypothetical vaccines in small populations of either heterosexual individuals [17] or MSM [34]. In accordance with our findings, they concluded that a vaccine of moderate level and duration of protection (60% for 10 years) could substantially reduce prevalence of infection by > 30% [17]. Our analysis is novel in considering the effect of vaccination on the scale of all MSM within a country, by comparing alternative realistic deployment strategies, and by incorporating the possible global emergence of a new extensively resistant strain, as well as incorporating a statistically rigorous fit to incidence data.

We considered scenarios in which sexual health services are able to meet the additional demand caused by increasing levels of gonorrhea incidence and antibiotic resistance. However, in recent years, access to UK sexual health services has worsened for individuals with symptoms of an acute sexually transmitted infection [35]. This insufficient capacity in sexual health clinics could create a vicious circle, where treatment delays cause onward transmission, increased incidence, and further unmet treatment need, a situation that would be exacerbated by antibiotic resistance [36, 37]. A gonococcal vaccine would offer important benefits of easing this pressure by averting infections, thereby reducing demand on clinics and avoiding the vicious circle.

There are 2 important areas for attention by public health researchers and policy-makers: first, the criteria on which treatment guidelines are formulated, and second, the need for well-designed vaccine trials that incorporate the aim of improving our understanding of the natural history of *N. gonorrhoeae*. Treatment guidelines are currently based on the proportion of diagnosed cases that are drug-resistant, with exceedance of a 5% threshold prompting changes to recommendations. However, it is important for policymakers also to consider the total number of resistant cases—especially in scenarios where resistant gonorrhea becomes more difficult and costlier to treat, as exemplified by a recent case of multidrug-resistant gonorrhea that required 3 days of inpatient treatment with intravenous ertapenem [38].

Trials of vaccine candidates need to be designed to address important gaps in knowledge, including the possibility of perverse outcomes. For example, if vaccination reduces the bacterial load, then this might reduce transmission by reducing infectivity. Alternatively, it might promote transmission if it reduces the probability or severity of symptoms and thereby increases the proportion of infections that are left untreated and hence persistent [32]. Not only could persistent infections lead to increased transmission, but they may increase the probability of drug resistance evolving within asymptomatic hosts [39]. Conversely, the resistance selection pressures could be reduced if fewer infections are being treated with antibiotics [18]. The relationship between determinants of drug resistance and antigenicity is not known and trials should monitor the diversity of lineages of *N. gonorrhoeae*. If a determinant of drug resistance is immunogenic and included in the vaccine, then this would enhance the beneficial impact of vaccination, while a vaccine that is more effective against drug-sensitive strains could increase the relative prevalence of resistance. Notwithstanding these interrogations and need for further research, a gonococcal vaccine could offer the hope of bringing the current epidemic of gonorrhea under control.

## Supplementary Material

ciz1241_suppl_Supplementary_Material

## Notes

Presented in part: International Society for Sexually Transmitted Disease Research Conference, Vancouver, Canada, 14–17 July 2019. Poster P630.

***Disclaimer*.** The study sponsors had no role in study design, data collection, data analysis, data interpretation, report writing, or decision to submit for publication. The views expressed are those of the authors and not necessarily those of the United Kingdom (UK) Department of Health and Social Care, the Department for International Development (DFID), the European Union (EU), the UK Medical Research Council (MRC), the National Health Service, the National Institute for Health Research (NIHR), or Public Health England (PHE). The corresponding author had full access to all study data and had final responsibility for the decision to submit for publication.

***Financial support.*** This work was supported by the MRC, Centre for Global Infectious Disease Analysis (grant number MR/R015600/1); this award is jointly funded by the MRC and DFID under the MRC/DFID Concordat agreement and is also part of the European and Developing Countries Clinical Trials Partnership (EDCTP2) programme supported by the EU. This work was also supported by the NIHR, Health Protection Research Unit in Modelling Methodology at Imperial College London, in partnership with PHE (grant number HPRU-2012–10080).

***Potential conflicts of interest.*** L. K. W. reports grants from the MRC outside the submitted work (work on Group A *Streptococcus*). P. J. W. reports grants from the NIHR outside the submitted work (funding to work on tuberculosis). X. D. reports grants from the MRC outside the submitted work (research grant for work on genomic epidemiology). All authors have submitted the ICMJE Form for Disclosure of Potential Conflicts of Interest. Conflicts that the editors consider relevant to the content of the manuscript have been disclosed.
