# Supplementary material for: Assessment of the Potential of Vaccination to Combat Antibiotic Resistance in Gonorrhea: A Modeling Analysis to Determine Preferred Product Characteristics
Source: Clin Infect Dis. 2020 Jan 6;71(8):1912–9. doi: 10.1093/cid/ciz1241 (PMC7643747; doi:10.1093/cid/ciz1241)
Supplement: ciz1241_suppl_Supplementary_Material [file ciz1241_suppl_supplementary_material.pdf]

Supplementary Information

Materials and methods

The numbers of annual diagnoses of gonorrhea in MSM in England between 2008 and 2017 were extracted from the surveillance data published annually by Public Health England [1]. England collects surveillance data for sexually transmitted infections (STI) via the genitourinary medicine clinic activity dataset (GUMCAD), which was introduced in 2008. GUMCAD is a mandatory statistical return, collected quarterly and published, in aggregate, annually. It contains data on all gonorrhea diagnoses made in specialist STI clinics, which account for the vast majority of gonorrhea diagnoses in England [2]. GUMCAD collects data on the gender, age, self-reported sexual orientation, and ethnicity of patients, as well as their country of birth and the location of the clinic attended. There have been no substantive changes to the GUMCAD surveillance practices of gonorrhea in MSM since its initiation.

We adapted a previously published stochastic compartmental model of gonorrhea [3] to allow for sexual heterogeneity by separating the population into two groups based on sexual activity levels $g \in \{H, L\}$ and adding vaccine protection . The full model is illustrated in Figure S1 with notations summarised in Supplementary Tables S1 and S2.

The initial population size was N = 600,000, with rates of population entry and exit calibrated based on data from the UK Office for National Statistics (Supplementary Figure S2) [3, 4]. The number of MSM in each activity group is denoted $N_g(t)$ $(N_L(t) + N_H(t) = N(t))$. Individuals remain in the sexual activity group to which they are initially assigned throughout the model simulations. The proportion of the population in each sexual activity group and average number of partners of those in each activity group were calibrated according to the Natsal-3 data. The 85% of MSM having up to five partners a year were assigned to the group L, with the remaining 15% who had more than five partners per year assigned to group H.

The rate of infection for uninfected individuals depends on the probability of infection per partnership (β), the average partner change rate of the activity group (p_g) and the sexual mixing pattern (ϵ), i.e the number of partnerships formed within and between activity groups. The parameter ϵ represents the sexual mixing coefficient and ranges from 0 to 1, representing a range of scenarios from proportionate mixing, where partners are chosen at random regardless of their sexual activity group, to assortative mixing, where partners are preferentially chosen from an individual's own sexual activity group [5].

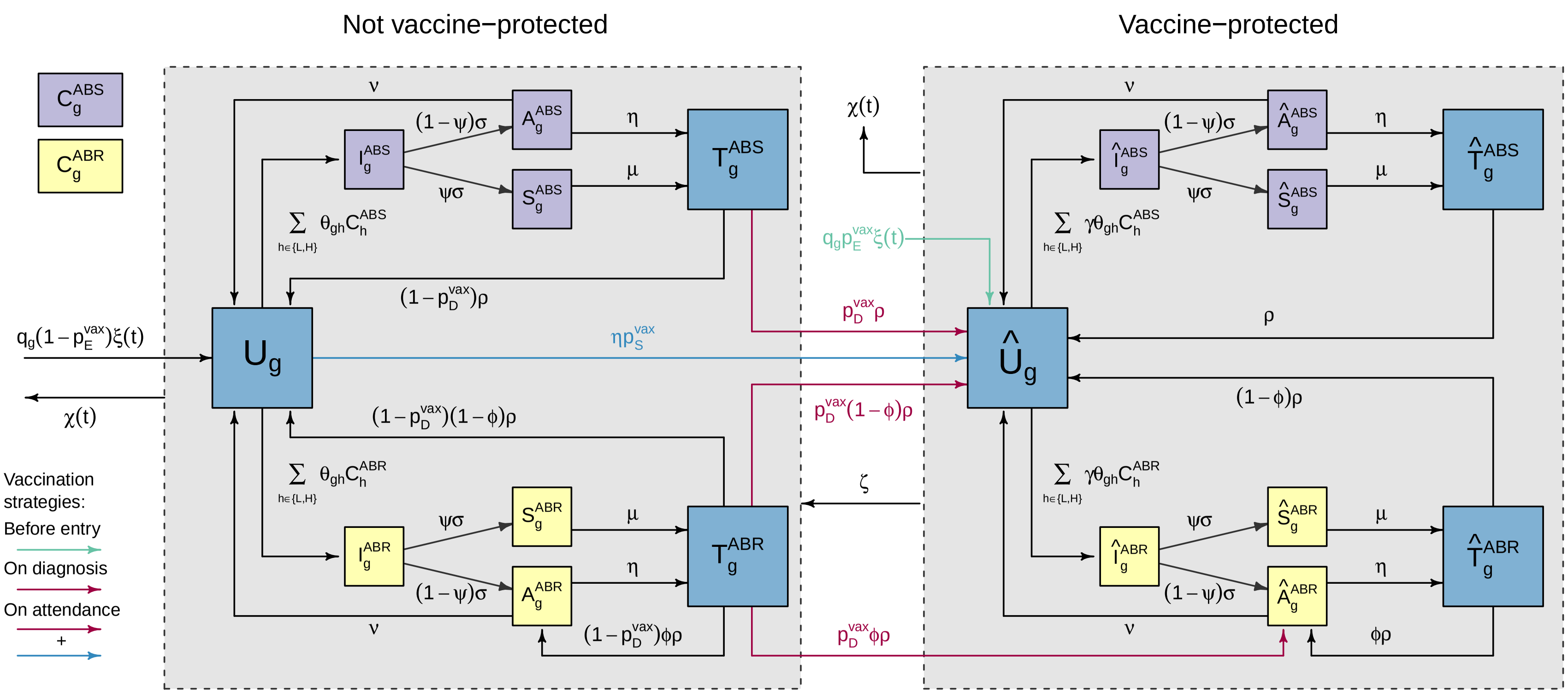

Figure S1. Model structure flow diagram. This figure corresponds to Figure 1 in the main paper, but with arrows labelled with algebraic expressions, as defined in Table S1.

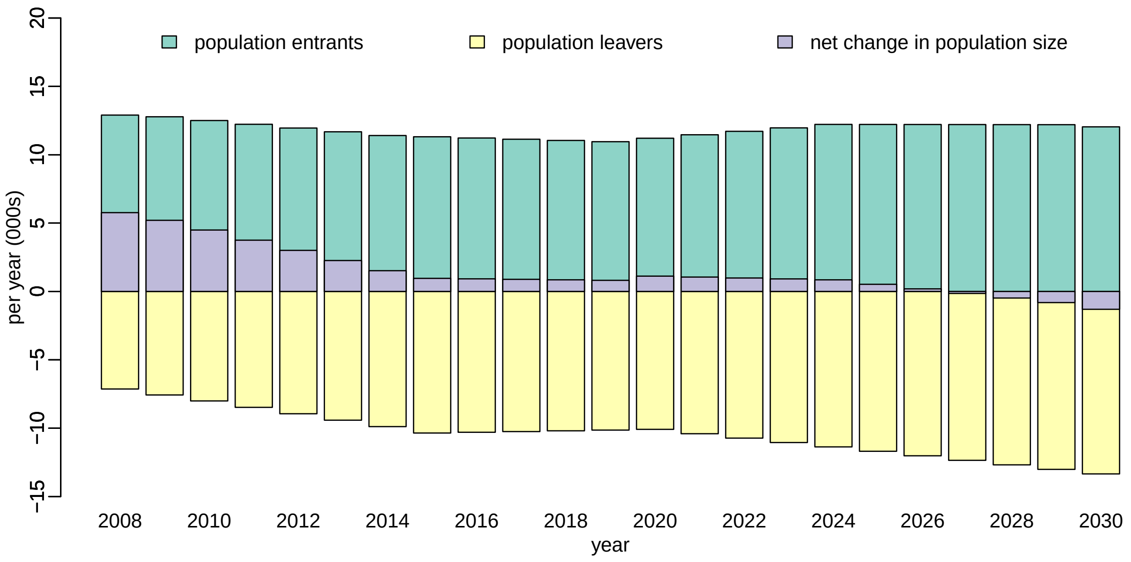

Figure S2. Expected number of population entrants, at age 15, and leavers, at age 65, between 2008 and 2030 derived from ONS population projections assuming 3.3% of men are MSM and assuming an initial population size of 600,000

Table S1. Input parameter notations, descriptions and values used in simulations

	Parameter description	Unit	Input value(s)
N_0	total population at t_0	#	600,000
q_L	proportion of population in group L	%	85
$\xi(t)$	rate of new MSM entering population	year^{-1}	Supplementary Figure S2
$\chi(t)$	per-capita rate of MSM leaving population	year^{-1}	Supplementary Figure S2
p_L	partner-change rate in group L	year^{-1}	0.6
p_H	partner-change rate in group H	year^{-1}	15.6
$p_E^{\text{vax}}(t)$	proportion of new entrants that are vaccinated	%	0-100
$p_D^{\text{vax}}(t)$	proportion of diagnosed MSM that are vaccinated	%	0-100
$p_S^{\text{vax}}(t)$	proportion of screened MSM that are vaccinated	%	0-100
$1/\zeta$	expected duration of vaccine protection	years	1-20
γ	efficacy: vaccine-conferred reduction in susceptibility to infection	%	1-100
ω	number of imported ABR cases	year^{-1}	10
ϕ	ABR infections for which treatment fails	%	0-100

The rate of transmission from an individual in group h to an uninfected individual in group g is denoted θ_{gh} and defined as follows:

$$\theta_{\mathrm{LL}}(t) = \frac{p_L \beta}{N_L(t)} \left(\epsilon + (1-\epsilon) \frac{p_L N_L(t)}{p_L N_L(t) + p_H N_H(t)} \right) \tag{S1}$$

$$\theta_{\mathrm{LH}}(t) = \frac{p_L \beta}{N_H(t)} (1-\epsilon) \frac{p_H N_H(t)}{p_L N_L(t) + p_H N_H(t)} = \frac{p_L p_H \beta (1-\epsilon)}{p_L N_L(t) + p_H N_H(t)} \tag{S2}$$

$$\theta_{\mathrm{HL}}(t) = \frac{p_H \beta}{N_L(t)} (1-\epsilon) \frac{p_L N_L(t)}{p_L N_L(t) + p_H N_H(t)} = \frac{p_L p_H \beta (1-\epsilon)}{p_L N_L(t) + p_H N_H(t)} \tag{S3}$$

$$\theta_{\mathrm{HH}}(t) = \frac{p_H \beta}{N_H(t)} \left(\epsilon + (1-\epsilon) \frac{p_H N_H(t)}{p_L N_L(t) + p_H N_H(t)} \right) \tag{S4}$$

Individuals in group g are initially uninfected (U_g). They become infected with strain $s \in \{\mathrm{ABS}, \mathrm{ABR}\}$, denoting antibiotic susceptible (ABS) and ABR strains respectively. The model assumes that strains do not vary in transmissibility, propensity to cause symptoms or rate of natural clearance, and that the rate of infection from a contagious individual in group h to an uninfected individual in group g is $\theta_{gh}(t)$. Infected individuals initially pass through an incubation period (I_g^s), which they leave at rate σ. A proportion ψ of those infected then go on to develop symptoms (S_g^s), whereas the rest remain asymptomatic (A_g^s). Symptomatic individuals (S_g^s) seek care at rate μ. Individuals without symptoms attend sexual health screening at rate η. Natural clearance of asymptomatic infection happens at rate ν. Treated individuals recover from the infection and become uninfected again at rate ρ, with the exception of a proportion ϕ of the individuals infected with an ABR strain (T_g^{ABR}) for whom treatment fails, leading to persistent infection for which the same dynamics are assumed as for asymptomatic cases. Gonococcal infection can occur in the rectum, pharynx and/or urethra, resulting in different rates of onward transmission and probabilities of developing symptoms [6]. Surveillance data are not stratified by infection site (rectum, pharynx, urethra), hence the parameters are averages across sites. The model assumes that no natural immunity is conferred by infection [7,8]. The ABR strain is imported into the highly sexually active group at rate $\omega/N_H(t)$ per person per year from time t_{ABR}=2020 onwards.

Interventions

We considered three potential vaccination strategies in the model:

- Vaccination immediately before entry into the population - where a proportion $p_E^{\mathrm{vax}} > 0$ of adolescents are vaccinated before they become sexually active;
- Vaccination on diagnosis - where the vaccine is given to a proportion $p_D^{\mathrm{vax}} > 0$ of MSM diagnosed with gonorrhea;
- Vaccination on attendance - where the vaccine is given to a proportion $p_S^{\mathrm{vax}} = p_D^{\mathrm{vax}} > 0$ of MSM that present to sexual health services, either through screening or due to symptoms.

We varied vaccine efficacy in the analysis, with factor γ denoting the relative reduction in susceptibility to gonorrhea conferred by the vaccine, which is known as degree-type protection [9]. As such, vaccinated MSM ($\hat{U}_g$) can still become infected, albeit less frequently, on sexual contact with contagious individuals (C). These vaccinated-yet-infected individuals progress through the stages of infection ($\hat{I}_g, \hat{S}_g, \hat{A}_g$) similarly to those who are unvaccinated. The contagious population for each strain s is denoted $C_g^s = I_g^s + S_g^s + A_g^s + \hat{I}_g^s + \hat{S}_g^s + \hat{A}_g^s$. Under the vaccination-on-diagnosis and vaccination-on-attendance strategies, treatment fails for a proportion ϕ of those infected with the ABR strain, who remain contagious,

despite receiving the vaccine. Vaccine protection wanes at rate ζ per year, after which time individuals become fully susceptible to infection once more.

Model equations and stochastic simulations

In order to illustrate the model dynamics, we describe a deterministic version of the model by the differential equations S5 - S20, which are easier to read than the stochastic specification of the model, which follows.

$$\begin{aligned}\frac{dU_H(t)}{dt} &= \big(1-p_E^{\text{vax}}(t)\big)q_H\xi(t) - \Big(\theta_{HL}C_L(t)+\theta_{HH}C_H(t)+\eta p_S^{\text{vax}}(t)+\frac{\omega(t)}{N_H(t)}+\chi(t)\Big)U_H(t) \\ &\quad + \nu\big(A_H^{\text{ABS}}(t)+A_H^{\text{ABR}}(t)\big) + \big(1-p_D^{\text{vax}}(t)\big)\rho\Big(T_H^{\text{ABS}}(t)+\big(1-\phi\big)T_H^{\text{ABR}}(t)\Big)+\zeta\hat{U}_H(t)\end{aligned} \tag{S5}$$

$$\begin{aligned}\frac{dU_L(t)}{dt} &= \big(1-p_E^{\text{vax}}(t)\big)q_L\xi(t) - \Big(\theta_{LL}C_L(t)+\theta_{LH}C_H(t)+\eta p_S^{\text{vax}}(t)+\chi(t)\Big)U_L(t) \\ &\quad + \nu\big(A_L^{\text{ABS}}(t)+A_L^{\text{ABR}}(t)\big) + \big(1-p_D^{\text{vax}}(t)\big)\rho\Big(T_L^{\text{ABS}}(t)+\big(1-\phi\big)T_L^{\text{ABR}}(t)\Big)+\zeta\hat{U}_L(t)\end{aligned} \tag{S6}$$

$$\frac{dI_H^{\text{ABR}}(t)}{dt} = \Big(\theta_{HL}C_L^{\text{ABR}}(t)+\theta_{HH}C_H^{\text{ABR}}(t)+\frac{\omega(t)}{N_H(t)}\Big)U_H(t) - \big(\sigma+\chi(t)\big)I_H^{\text{ABR}}(t)+\zeta\hat{I}_H^{\text{ABR}}(t) \tag{S7}$$

$$\frac{dI_g^s(t)}{dt} = \Big(\theta_{gL}C_L^s(t)+\theta_{gH}C_H^s(t)\Big)U_g(t) - \big(\sigma+\chi(t)\big)I_g^s(t)+\zeta\hat{I}_g^s(t) \quad \text{otherwise} \tag{S8}$$

$$\frac{dS_g^s(t)}{dt} = \psi\sigma I_g^s(t) - \big(\mu+\chi(t)\big)S_g^s(t)+\zeta\hat{S}_g^s(t) \tag{S9}$$

$$\frac{dA_g^{\text{ABS}}(t)}{dt} = (1-\psi)\sigma I_g^{\text{ABS}}(t) - \big(\nu+\eta+\chi(t)\big)A_g^{\text{ABS}}(t)+\zeta\hat{A}_g^{\text{ABS}}(t) \tag{S10}$$

$$\frac{dA_g^{\text{ABR}}(t)}{dt} = (1-\psi)\sigma I_g^{\text{ABR}}(t) - \big(\nu+\eta+\chi(t)\big)A_g^{\text{ABR}}(t)+\big(1-p_D^{\text{vax}}(t)\big)\phi\rho T_g^{\text{ABR}}(t)+\zeta\hat{A}_g^{\text{ABR}}(t) \tag{S11}$$

$$\frac{dT_g^s(t)}{dt} = \mu S_g^s(t)+\eta A_g^s(t) - \big(\rho+\chi(t)\big)T_g^s(t)+\zeta\hat{T}_g^s(t) \tag{S12}$$

$$\begin{aligned}\frac{d\hat{U}_H(t)}{dt} &= p_E^{\text{vax}}(t)q_H\xi(t)+\eta p_S^{\text{vax}}(t)U_H(t)+p_D^{\text{vax}}(t)\rho\Big(T_H^{\text{ABS}}(t)+\big(1-\phi\big)T_H^{\text{ABR}}(t)\Big) \\ &\quad - \Big(\gamma\Big(\theta_{HL}C_L(t)+\theta_{HH}C_H(t)+\frac{\omega(t)}{N_H(t)}\Big)+\zeta+\chi(t)\Big)\hat{U}_H(t) \\ &\quad + \nu\Big(\hat{A}_H^{\text{ABS}}(t)+\hat{A}_H^{\text{ABR}}(t)\Big)+\rho\Big(\hat{T}_H^{\text{ABS}}(t)+\big(1-\phi\big)\hat{T}_H^{\text{ABR}}(t)\Big)\end{aligned} \tag{S13}$$

$$
\begin{aligned}
\frac{d\hat{U}_L(t)}{dt} &= p_E^{\mathrm{vax}}(t) q_L \xi(t) + \eta p_S^{\mathrm{vax}}(t) U_L(t) + p_D^{\mathrm{vax}}(t)\rho\Big(T_L^{\mathrm{ABS}}(t) + \big(1-\phi\big)T_L^{\mathrm{ABR}}(t)\Big) \\
&- \Big(\gamma\big(\theta_{LL}C_L(t) + \theta_{LH}C_H(t)\big) + \zeta + \chi(t)\Big)\hat{U}_L(t) \\
&+ \nu\Big(\hat{A}_L^{\mathrm{ABS}}(t) + \hat{A}_L^{\mathrm{ABR}}(t)\Big) + \rho\Big(\hat{T}_L^{\mathrm{ABS}}(t) + \big(1-\phi\big)\hat{T}_L^{\mathrm{ABR}}(t)\Big)
\end{aligned} \tag{S14}
$$

$$
\frac{d\hat{I}_H^{\mathrm{ABR}}(t)}{dt} = \gamma\left(\theta_{HL}C_L^{\mathrm{ABR}}(t) + \theta_{HH}C_H^{\mathrm{ABR}}(t) + \frac{\omega(t)}{N_H(t)}\right)\hat{U}_H(t) - \Big(\sigma + \zeta + \chi(t)\Big)\hat{I}_H^{\mathrm{ABR}}(t) \tag{S15}
$$

$$
\frac{d\hat{I}_g^s(t)}{dt} = \gamma\Big(\theta_{gL}C_L^s(t) + \theta_{gH}C_H^s(t)\Big)\hat{U}_g(t) - \Big(\sigma + \zeta + \chi(t)\Big)\hat{I}_g^s(t) \quad \text{otherwise} \tag{S16}
$$

$$
\frac{d\hat{S}_g^s(t)}{dt} = \psi\sigma\hat{I}_g^s(t) - \big(\mu + \zeta + \chi(t)\big)\hat{S}_g^s(t) \tag{S17}
$$

$$
\frac{d\hat{A}_g^{\mathrm{ABS}}(t)}{dt} = \big(1-\psi\big)\sigma\hat{I}_g^{\mathrm{ABS}}(t) - \big(\nu + \eta + \zeta + \chi(t)\big)\hat{A}_g^{\mathrm{ABS}}(t) \tag{S18}
$$

$$
\frac{d\hat{A}_g^{\mathrm{ABR}}(t)}{dt} = p_D^{\mathrm{vax}}\phi\rho T_g^{\mathrm{ABR}}(t) + \big(1-\psi\big)\sigma\hat{I}_g^{\mathrm{ABR}}(t) - \big(\nu + \eta + \zeta + \chi(t)\big)\hat{A}_g^{\mathrm{ABR}}(t) + \phi\rho\hat{T}_g^{\mathrm{ABR}}(t) \tag{S19}
$$

$$
\frac{d\hat{T}_g^s(t)}{dt} = \mu\hat{S}_g^s(t) + \eta\hat{A}_g^s(t) - \big(\rho + \zeta + \chi(t)\big)\hat{T}_g^s(t) \tag{S20}
$$

We used a stochastic version of the model described in Equations S5 - S20, time-discretised using the Gillespie tau-leap method with steps of 1 day. The process was initialised on 31 December 2007, with $A_g^{\mathrm{ABS}}(0)$ asymptomatic infections in each sexual activity group $g \in \{L, H\}$ and the remainder of the population uninfected. Simulation proceeded through iterations of the steps below for each day. Since the model is stochastic, the outbreak can become extinct even when its basic reproduction number is >1. Transition variables $b_1^g, b_2^g, d_1^g, \ldots, d_{32}^g$ are drawn from the following distributions:

$$b_1^g \sim \mathrm{Poisson}\Big(q_g\big(1-p_E^{\mathrm{vax}}\big)\xi(t)\Big) \tag{S21}$$

$$b_2^g \sim \mathrm{Poisson}\Big(q_g p_E^{\mathrm{vax}}\xi(t)\Big) \tag{S22}$$

$$\begin{aligned} d_1^H, \ldots, d_4^H \sim \mathrm{Multinom}\Bigg(&U_H(t),\ \theta_{HL}C_L^{\mathrm{ABS}}(t) + \theta_{HH}C_H^{\mathrm{ABS}}(t), \\ &\theta_{HL}C_L^{\mathrm{ABR}}(t) + \theta_{HH}C_H^{\mathrm{ABR}}(t) + \frac{\omega(t)}{N_H(t)},\ \eta p_S^{\mathrm{vax}}(t),\ \chi(t)\Bigg) \end{aligned} \tag{S23}$$

$$\begin{aligned} d_1^L, \ldots, d_4^L \sim \mathrm{Multinom}\Bigg(&U_L(t),\ \theta_{LL}C_L^{\mathrm{ABS}}(t) + \theta_{LH}C_H^{\mathrm{ABS}}(t), \\ &\theta_{LL}C_L^{\mathrm{ABR}}(t) + \theta_{LH}C_H^{\mathrm{ABR}}(t),\ \eta p_S^{\mathrm{vax}}(t),\ \chi(t)\Bigg) \end{aligned} \tag{S24}$$

$$d_5^g, d_6^g, d_7^g \sim \mathrm{Multinom}\Big(I_g^{\mathrm{ABS}}(t),\ \psi\sigma,\ \big(1-\psi\big)\sigma,\ \chi(t)\Big) \tag{S25}$$

$$d_8^g, d_9^g, d_{10}^g \sim \mathrm{Multinom}\Big(I_g^{\mathrm{ABR}}(t),\ \psi\sigma,\ \big(1-\psi\big)\sigma,\ \chi(t)\Big) \tag{S26}$$

$$d_{11}^g, d_{12}^g \sim \mathrm{Multinom}\big(S_g^{\mathrm{ABS}}(t),\ \mu,\ \chi(t)\big) \tag{S27}$$

$$d_{13}^g, d_{14}^g \sim \mathrm{Multinom}\big(S_g^{\mathrm{ABR}}(t),\ \mu,\ \chi(t)\big) \tag{S28}$$

$$d_{15}^g, d_{16}^g, d_{17}^g \sim \mathrm{Multinom}\big(A_g^{\mathrm{ABS}}(t),\ \nu,\ \eta,\ \chi(t)\big) \tag{S29}$$

$$d_{18}^g, d_{19}^g, d_{20}^g \sim \mathrm{Multinom}\big(A_g^{\mathrm{ABR}}(t),\ \nu,\ \eta,\ \chi(t)\big) \tag{S30}$$

$$d_{21}^g, d_{22}^g, d_{23}^g \sim \mathrm{Multinom}\Big(T_g^{\mathrm{ABS}}(t),\ \big(1-p_D^{\mathrm{vax}}\big)\rho,\ p_D^{\mathrm{vax}}\rho,\ \chi(t)\Big) \tag{S31}$$

$$d_{24}^g, \ldots, d_{28}^g \sim \mathrm{Multinom}\Big(T_g^{\mathrm{ABR}}(t),\ \big(1-p_D^{\mathrm{vax}}\big)\big(1-\phi\big)\rho,\ \big(1-p_D^{\mathrm{vax}}\big)\phi\rho,\ p_D^{\mathrm{vax}}\big(1-\phi\big)\rho,\ p_D^{\mathrm{vax}}\phi\rho,\ \chi(t)\Big) \tag{S32}$$

$$\begin{aligned} d_{29}^H, \ldots, d_{32}^H \sim \mathrm{Multinom}\Bigg(&\hat{U}_H(t),\ \gamma\Big(\theta_{HL}C_L^{\mathrm{ABS}}(t) + \theta_{HH}C_H^{\mathrm{ABS}}(t)\Big), \\ &\gamma\Big(\theta_{HL}C_L^{\mathrm{ABR}}(t) + \theta_{HH}C_H^{\mathrm{ABR}}(t) + \frac{\omega(t)}{N_H(t)}\Big),\ \zeta,\ \chi(t)\Bigg) \end{aligned} \tag{S33}$$

$$\begin{aligned} d_{29}^L, \ldots, d_{32}^L \sim \mathrm{Multinom}\Bigg(&\hat{U}_L(t),\ \gamma\Big(\theta_{LL}C_L^{\mathrm{ABS}}(t) + \theta_{LH}C_H^{\mathrm{ABS}}(t)\Big), \\ &\gamma\Big(\theta_{LL}C_L^{\mathrm{ABR}}(t) + \theta_{LH}C_H^{\mathrm{ABR}}(t)\Big),\ \zeta,\ \chi(t)\Bigg) \end{aligned} \tag{S34}$$

$$d_{33}^g, \ldots d_{36}^g \sim \mathrm{Multinom}\Big(\hat{I}_g^{\mathrm{ABS}}(t),\ \psi\sigma,\ \big(1-\psi\big)\sigma,\ \zeta,\ \chi(t)\Big) \tag{S35}$$

$$d_{37}^g, \ldots, d_{40}^g \sim \mathrm{Multinom}\Big(\hat{I}_g^{\mathrm{ABR}}(t),\ \psi\sigma,\ \big(1-\psi\big)\sigma,\ \zeta,\ \chi(t)\Big) \tag{S36}$$

$$d_{41}^g, d_{42}^g, d_{43}^g \sim \mathrm{Multinom}\big(\hat{S}_g^{\mathrm{ABS}}(t),\ \mu,\ \zeta,\ \chi(t)\big) \tag{S37}$$

$$d_{44}^g, d_{45}^g, d_{46}^g \sim \mathrm{Multinom}\big(\hat{S}_g^{\mathrm{ABR}}(t),\ \mu,\ \zeta,\ \chi(t)\big) \tag{S38}$$

$$d_{47}^g, \ldots, d_{50}^g \sim \mathrm{Multinom}\big(\hat{A}_g^{\mathrm{ABS}}(t),\ \nu,\ \eta,\ \zeta,\ \chi(t)\big) \tag{S39}$$

$$d_{51}^g, \ldots, d_{54}^g \sim \mathrm{Multinom}\big(\hat{A}_g^{\mathrm{ABR}}(t),\ \nu,\ \eta,\ \zeta,\ \chi(t)\big) \tag{S40}$$

$$d_{55}^g, d_{56}^g, d_{57}^g \sim \mathrm{Multinom}\Big(\hat{T}_g^{\mathrm{ABS}}(t),\ \rho,\ \zeta,\ \chi(t)\Big) \tag{S41}$$

$$d_{58}^g, \ldots, d_{61}^g \sim \mathrm{Multinom}\Big(\hat{T}_g^{\mathrm{ABR}}(t),\ \big(1-\phi\big)\rho,\ \phi\rho,\ \zeta,\ \chi(t)\Big) \tag{S42}$$

For ease of notation, we have expressed the equations above using the rates we have previously defined, rather than their respective probabilities. Therefore, for example, $d \sim \mathrm{Multinom}(\mathrm{S}, \underline{\mathrm{r}})$ should be understood as $d \sim \mathrm{Multinom}(\mathrm{S}, 1 - \mathrm{e}^{-\underline{\mathrm{r}}})$.

The compartments of the model were updated as follows:

$$U_g(t+1) := U_g(t) + b_1^g - d_1^g - d_2^g - d_3^g - d_4^g + d_{15}^g + d_{18}^g + d_{21}^g + d_{24}^g + d_{31}^g \quad \text{(S43)}$$

$$I_g^{\mathrm{ABS}}(t+1) := I_g^{\mathrm{ABS}}(t) + d_1^g - d_5^g - d_6^g - d_7^g + d_{35}^g \quad \text{(S44)}$$

$$I_g^{\mathrm{ABR}}(t+1) := I_g^{\mathrm{ABR}}(t) + d_2^g - d_8^g - d_9^g - d_{10}^g + d_{39}^g \quad \text{(S45)}$$

$$S_g^{\mathrm{ABS}}(t+1) := S_g^{\mathrm{ABS}}(t) + d_5^g - d_{11}^g - d_{12}^g + d_{42}^g \quad \text{(S46)}$$

$$S_g^{\mathrm{ABR}}(t+1) := S_g^{\mathrm{ABR}}(t) + d_8^g - d_{13}^g - d_{14}^g + d_{45}^g \quad \text{(S47)}$$

$$A_g^{\mathrm{ABS}}(t+1) := A_g^{\mathrm{ABS}}(t) + d_6^g - d_{15}^g - d_{16}^g - d_{17}^g + d_{49}^g \quad \text{(S48)}$$

$$A_g^{\mathrm{ABR}}(t+1) := A_g^{\mathrm{ABR}}(t) + d_9^g - d_{18}^g - d_{19}^g - d_{20}^g + d_{25}^g + d_{53}^g \quad \text{(S49)}$$

$$T_g^{\mathrm{ABS}}(t+1) := T_g^{\mathrm{ABS}}(t) + d_{11}^g + d_{16}^g - d_{21}^g - d_{22}^g - d_{23}^g + d_{56}^g \quad \text{(S50)}$$

$$T_g^{\mathrm{ABR}}(t+1) := T_g^{\mathrm{ABR}}(t) + d_{13}^g + d_{19}^g - d_{24}^g - d_{25}^g - d_{26}^g - d_{27}^g - d_{28}^g + d_{60}^g \quad \text{(S51)}$$

$$\hat{U}_g(t+1) := \hat{U}_g(t) + b_2^g + d_3^g + d_{22}^g + d_{26}^g - d_{29}^g - d_{30}^g - d_{31}^g - d_{32}^g + d_{47}^g + d_{51}^g + d_{55}^g + d_{58}^g \quad \text{(S52)}$$

$$\hat{I}_g^{\mathrm{ABS}}(t+1) := \hat{I}_g^{\mathrm{ABS}}(t) + d_{29}^g - d_{33}^g - d_{34}^g - d_{35}^g - d_{36}^g \quad \text{(S53)}$$

$$\hat{I}_g^{\mathrm{ABR}}(t+1) := \hat{I}_g^{\mathrm{ABR}}(t) + d_{30}^g - d_{37}^g - d_{38}^g - d_{39}^g - d_{40}^g \quad \text{(S54)}$$

$$\hat{S}_g^{\mathrm{ABS}}(t+1) := \hat{S}_g^{\mathrm{ABS}}(t) + d_{33}^g - d_{41}^g - d_{42}^g - d_{43}^g \quad \text{(S55)}$$

$$\hat{S}_g^{\mathrm{ABR}}(t+1) := \hat{S}_g^{\mathrm{ABR}}(t) + d_{37}^g - d_{44}^g - d_{45}^g - d_{46}^g \quad \text{(S56)}$$

$$\hat{A}_g^{\mathrm{ABS}}(t+1) := \hat{A}_g^{\mathrm{ABS}}(t) + d_{34}^g - d_{47}^g - d_{48}^g - d_{49}^g - d_{50}^g \quad \text{(S57)}$$

$$\hat{A}_g^{\mathrm{ABR}}(t+1) := \hat{A}_g^{\mathrm{ABR}}(t) + d_{27}^g + d_{38}^g - d_{51}^g - d_{52}^g - d_{53}^g - d_{54}^g + d_{59}^g \quad \text{(S58)}$$

$$\hat{T}_g^{\mathrm{ABS}}(t+1) := \hat{T}_g^{\mathrm{ABS}}(t) + d_{41}^g + d_{48}^g - d_{55}^g - d_{56}^g - d_{57}^g \quad \text{(S59)}$$

$$\hat{T}_g^{\mathrm{ABR}}(t+1) := \hat{T}_g^{\mathrm{ABR}}(t) + d_{44}^g + d_{52}^g - d_{58}^g - d_{59}^g - d_{60}^g - d_{61}^g \quad \text{(S60)}$$

Bayesian Inference

We considered the recorded cases ($Y_D(t)$, t = 2008, ..., 2017) as the observed realisations of an underlying unobserved Markov process: the total number of diagnosed gonococcal infections ($Z_D(t)$) (Supplementary Figure S3). We allowed for a 10% margin of under-reporting, such that 90% of all gonorrhea diagnoses are recorded in the surveillance data - consistent with evidence of the proportion of gonorrhea diagnoses made in a General Practice setting and therefore not reported [2].

$$Y_D(t) \sim \mathcal{N}(0.9Z_D(t), 0.05Z_D(t)) \quad \text{(S61)}$$

Additionally, to ensure that the rate at which MSM attend sexual health screening reflects the most recent data, we considered the number of sexual health clinic attendances in 2017 ($Y_A(t)$, t = 2017) as the observed realisation of an underlying unobserved variable - the total number of MSM attending sexual health services ($Z_A(2017)$), again allowing for a 10% margin of under-reporting.

$$Y_A(2017) \sim \mathrm{Poisson}(0.9Z_A(2017)) \quad \text{(S62)}$$

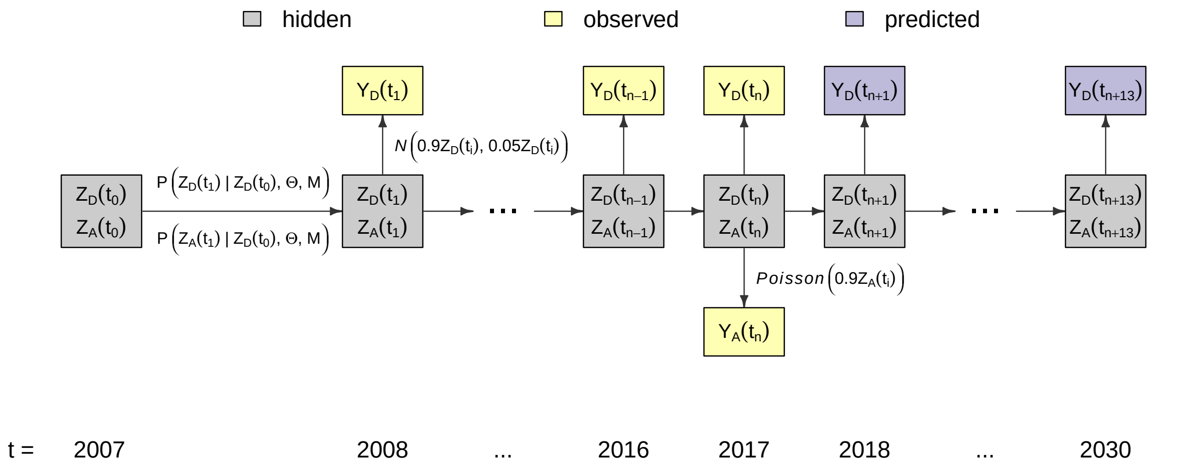

Figure S3. Illustrative diagram of the partially observed Markov process The total incidence of gonorrhea infections, $Z_D(t)$, and annual sexual health clinic attendances, $Z_A(t)$ are unobserved Markovian time series, updated via the model of infection, M, with parameters Θ. The number of recorded cases, $Y_D(t)$, and recorded attendances, $Y_A(t)$ are the observed realisations of the underlying hidden processes.

An expression for the likelihood of the observed data given the model is not analytically tractable, so we used a particle filter to compute an unbiased estimate. This estimated likelihood was then incorporated into a Monte Carlo Markov Chain (MCMC) algorithm, which was used to sample from the joint posterior distribution of the parameters given the observed data [10].

As the number of parameters increases, so does the difficulty of exploring the joint posterior space. To be parsimonious we reduced the number of model parameters by making the initial number of symptomatic cases, $S_L(2007)$ and $S_H(2007)$, to be zero. After a few simulated days these variables quickly reach the stochastic values implied by the model and parameters. Therefore, the first few days of each simulation can be thought of as the equivalent to a Markov Chain convergence or burn-in step.

We implemented the model fitting using a pMCMC algorithm from the R package pomp [11], which we modified to enable parallel computation. The estimation of the likelihood was based on 1,000 particles, which we found to be sufficiently robust to produce consistent estimates of the likelihood. Four pMCMC with dispersed starting points were run for 110,000 iterations, with the first 10% discarded as burn-in. The chains were compared using the R package coda [12]; the multivariate Gelman-Rubin diagnostic was $\leq$1.1 for all inferred parameters, suggesting that they appear to have converged on the same posterior distribution [13]. For robustness, we combined the posterior samples from all four chains and ensured all parameters had an effective sample size greater than 200.

Prior distributions of parameters

Performing Bayesian inference requires the specification of plausible parameter priors. For parameters whose values are uncertain ($\epsilon, \frac{A_L^{\text{ABS}}(0)}{N_L}, \frac{A_H^{\text{ABS}}(0)}{N_H}, \beta, \psi$) we adopted highly uninformative uniform priors (Table S2). The remaining four parameters ν, σ, μ and ρ were assigned informative priors based on literature review, as summarised in Table S2.

The length of time for which asymptomatic gonorrhea infection persists has not been well measured. Duration of carriage likely depends on the site of infection, with estimates varying widely from 12 weeks at the pharynx, up to a year for rectal infection [14, 15]. In a study of 18 asymptomatic men infected with (urethral) gonorrhea, no infections were cleared in the 165 days from diagnosis to treatment [16]. Modelling studies commonly use an average duration of untreated infection of 6 months [5, 17, 18]. This is supported by evidence from genomic data, where bacterial genomes isolated from known contact pairs had a maximum time to most recent common ancestor of 8 months [19]. We therefore assigned the duration of untreated carriage, $\frac{1}{\nu}$, a prior corresponding to a mean duration of carriage between three months and one year with 95% prior weight.

The UK national guidelines on safer sex advice recommend MSM test for gonorrhea annually, and that highly sexually active MSM get tested every three months [20, 21]. However, in a social media survey of 2,668 MSM in Scotland, Wales and Ireland, two-thirds reported STI testing less than once a year [22]. We assigned a uniform distribution to the rate of screening, η with 95% prior weight between 0.4 and 4. This corresponds to a range of scenarios: from testing at quarterly intervals, in adherence with the British Association for Sexual Health and HIV guidelines, through to the situation described by [22]. The lower bound for η was calculated by setting the probability of testing less than once a year equal to $\frac{2}{3}$ under an exponential distribution with rate η, so that $\mathcal{P}[T > 1] = e^{-\eta} = \frac{2}{3}$, and solving to find $\eta = -\ln \frac{2}{3} = 0.4$.

The length of time spent in the incubation, symptomatic, and treatment stages of infection are observed to be short [23–25]. We set priors accordingly: the mean duration of incubation, $\frac{1}{\sigma}$, was between three and eight days; the mean duration of symptomatic infection (before seeking care), $\frac{1}{\mu}$, was between a day and two weeks; and the mean duration of treatment, $\frac{1}{\rho}$, was between five and nine days, in accordance with the BASHH recommendation to abstain from sex for seven days after treatment [26].

Table S2. Calibrated parameter notations, descriptions, prior and posterior distributions

	Parameter description	Unit	Prior distribution	Prior mean (95% CI)	Posterior mean (95% CrI)
ϵ	level of assortativity in sexual mixing	-	$\mathcal{U}[0,1]$	0.5 (0.025, 0.975)	0.43 (0.06, 0.74)
$\frac{A_L^{\text{ABS}}(0)}{N_L}$	asymptomatic prevalence in group L at t_0	%	$\mathcal{U}[0,10]$	5 (0.25, 9.75)	0.9 (0.2, 1.7)
$\frac{A_H^{\text{ABS}}(0)}{N_H}$	asymptomatic prevalence in group H at t_0	%	$\mathcal{U}[0,10]$	5 (0.25, 9.75)	1.0 (0.4, 1.7)
β	probability of transmission per partnership	%	$\mathcal{U}[0,100]$	50 (2.5, 97.5)	58.5 (28.3, 96.5)
ψ	infections that become symptomatic	%	$\mathcal{U}[0,100]$	50 (2.5, 97.5)	24.5 (13.3, 35.2)
$1/\sigma$	average duration of incubation period	days	$\text{InvGamma}(17, 4.6)$	4.7 (3.0, 8.0)	4.6 (3.0, 7.6)
$1/\nu$	average duration of untreated asymptomatic carriage	days	$\text{InvGamma}(8, 0.29)$	157 (87, 364)	174 (95, 443)
η	rate of screening when asymptomatic	year^{-1}	$\text{U}[0.4, 4]$	2.2 (0.49, 3.91)	0.45 (0.44, 0.46)
$1/\mu$	average time to symptomatics seeking care	days	$\text{InvGamma}(3, 45.6)$	2.7 (1.1, 12.9)	2.8 (1.2, 14.7)
$1/\rho$	average time to recovery following treatment	days	$\text{InvGamma}(101, 0.52)$	7.0 (5.8, 8.5)	6.9 (5.8, 8.6)

Estimation of model parameters

The posterior estimate of the probability of transmission per partnership per year β was 59% with a wide range of credible values from 28% to 97%. The parameter was positively correlated with the level of assortativity in sexual mixing ϵ, which was estimated to be between 0.06 and 0.75 with 95% credibility. This corresponds to the trade-off required to maintain a force of infection compatible with the observed data, as described by equations S1-4.

The model predicts that 25% (95% CrI: 13%, 35%) of infections become symptomatic, and that this proportion (ψ) is strongly negatively correlated with the rate of asymptomatic screening (η). This corresponds to the tradeoff between duration of carriage, $\frac{1}{\nu+\eta}$, and the proportion of infections entering the carriage state $(1-\psi)$

The parameters corresponding to the time spent in the incubation, symptomatic, and treatment stages of infection (σ, μ, ρ) had posterior distributions that closely matched their prior distributions, suggesting that the priors were appropriate and that there is little additional information on these parameters to be gleaned from this data set. These parameters did not show a strong correlation with any others; as expected given the short duration of the incubation, symptomatic and treatment stages variation in their parameters makes little difference to the dynamics of infection.

The posterior distribution of ν has a slightly lower mean than the prior, implying a longer mean duration of carriage: 174 (95% CrI: 95, 443) days compared to 157 (95% CrI: 87, 364). The prior and posterior credible intervals intersect to a large extent so there is not significant evidence of a departure from the prior based on the data.

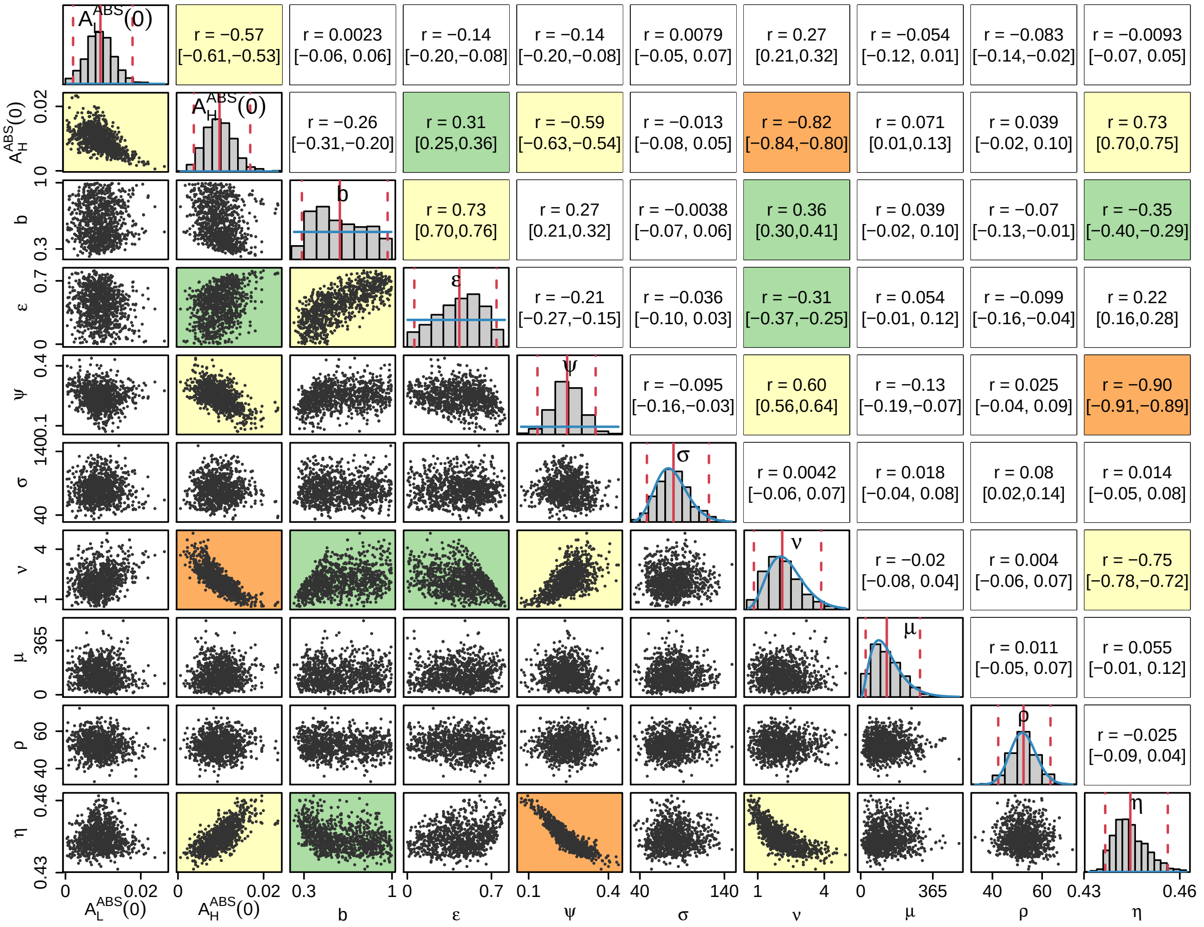

Figure S4. Posterior distributions of parameters. The diagonal plots show histograms of the posterior distributions for all sampled parameters. The blue lines show prior distributions, and the red lines indicate posterior mean and 95% credible intervals. The plots below the diagonal show scatter plots based on 1,000 samples from the posterior, illustrating the relationships between pairs of estimated parameters. An orange background indicates a correlation higher than 0.8, a yellow background indicates a correlation between 0.5 and 0.8, a green background indicates a correlation between 0.3 and 0.5, and a white background indicates a correlation less than 0.3. The plots above the diagonal show the corresponding correlation coefficients with the 95% credible intervals in parentheses.

Overview of simulation analysis

To examine the effect of vaccine efficacy and duration of protection, and the effect of different deployment strategies, we considered 1,000 vaccine profiles (i.e. combinations of level and duration of protection), selected using Latin Hypercube Sampling, which ranged in efficacy from 1-100% and offered protection lasting between 1 and 20 years. For each vaccine profile we performed 100 Monte Carlo simulations of the epidemic between 2008 and 2030. we repeated the analysis for each of the ABR strains of varying controllability, under each of the three vaccination strategies.

We assessed the potential impact of a MeNZB-like vaccine by considering 100 vaccine profiles conferring protection from infection ranging from 21%-39% with an assumed duration of two to four years. We repeated the analysis for varying levels of vaccine coverage to assess how the level of uptake achieved

impacts the success of each strategy.

References

1. Savage EJ, Mohammed H, Leong G, Duffell S, Hughes G. Improving surveillance of sexually transmitted infections using mandatory electronic clinical reporting: the genitourinary medicine clinic activity dataset, England, 2009 to 2013. Eurosurveillance. 2014;19(48):20981.
2. Wetten S, Mohammed H, Yung M, Mercer CH, Cassell JA, Hughes G. Diagnosis and treatment of chlamydia and gonorrhoea in general practice in England 2000 – 2011: a population-based study using data from the UK Clinical Practice Research Datalink. BMJ Open. 2015;5(e007776):1–9.
3. Whittles LK, White PJ, Didelot X. Estimating the fitness cost and benefit of cefixime resistance in Neisseria gonorrhoeae to inform prescription policy: a modelling study. PLoS Medicine. 2017;14(10):1–20.
4. Office for National Statistics. United Kingdom population mid-year estimate; 2016. Available from: `www.ons.gov.uk/peoplepopulationandcommunity/populationandmigration/populationestimates/timeseries/ukpop/pop`.
5. Garnett GP, Mertz KJ, Finelli L, Levine WC, St Louis ME. The transmission dynamics of gonorrhoea: modelling the reported behaviour of infected patients from Newark, New Jersey. Philosophical Transactions of the Royal Society of London Series B: Biological Sciences. 1999 apr;354:787–797.
6. Platt R, Rice P, McCormack W. Risk of acquiring gonorrhea and prevalence of abnormal adnexal findings among women recently exposed to gonorrhea. JAMA. 1983;250(23):3205–3209.
7. Schmidt KA, Schneider H, Lindstrom JA, Boslego JW, Warren RA, Van de Verg L, et al. Homologous gonococci in male volunteers. Sexually Transmitted Diseases. 2001;28(10):555–564.
8. Hosenfeld CB, Workowski KA, Berman S, Zaidi A, Dyson J, Mosure D, et al. Repeat infection with chlamydia and gonorrhea among females: a systematic review of the literature. Sexually Transmitted Diseases. 2009;36(8):478–489.
9. McLean AR, Blower SM. Imperfect vaccines and herd immunity to HIV. Proceedings of the Royal Society B: Biological Sciences. 1993;253:9–13.
10. Andrieu C, Doucet A, Holenstein R. Particle Markov chain Monte Carlo methods. Journal of the Royal Statistical Society Series B: Statistical Methodology. 2010;72(3):269–342.
11. King AA, Nguyen D, Ionides EL. Statistical inference for partially observed Markov processes via the R Package pomp. Journal of Statistical Software. 2016;69(12):1–43.
12. Plummer M, Best N, Cowles K, Vines K. CODA: convergence diagnosis and output analysis for MCMC. R News. 2006;6(1):7–11.
13. Brooks SPB, Gelman AG. General methods for monitoring convergence of iterative simulations. Journal of Computational and Graphical Statistics. 1998;7(4):434–455.
14. Fairley CK, Chen MY, Bradshaw CS, Tabrizi SN. Is it time to move to nucleic acid amplification tests screening for pharyngeal and rectal gonorrhoea in men who have sex with men to improve gonorrhoea control? Sexual Health. 2011;8(1):9–11.

15. Jin F, Prestage GP, Mao L, Kippax SC, Pell CM, Donovan B, et al. Incidence and risk factors for urethral and anal gonorrhoea and chlamydia in a cohort of HIV-negative homosexual men: the Health in Men Study. Sexually Transmitted Infections. 2007;83(2):113–119.

16. Handsfield HH, Lipman TO, Harnisch JP, Tronca E, Holmes KK. Asymptomatic gonorrhea in men. New England Journal of Medicine. 1974;290(3):117–123.

17. Yorke JA, Hethcote HW, Nold A. Dynamics and control of the transmission of gonorrhea. Sexually Transmitted Diseases. 1978;5(2):51–56.

18. White PJ, Ward H, Cassell JA, Mercer CH, Garnett GP. Vicious and virtuous circles in the dynamics of infectious disease and the provision of health care: gonorrhea in Britain as an example. The Journal of Infectious Diseases. 2005;192:824–836.

19. Didelot X, Dordel J, Whittles LK, Collins C, Bilek N, Bishop CJ, et al. Genomic analysis and comparison of two gonorrhoea outbreaks. mBio. 2016;7(3):e00525–16.

20. Clutterbuck D, Flowers P, Barber T, Wilson H, Nelson M, Hedge B, et al. The 2010 UK national guidelines on safer sex advice. The Clinical Effectiveness Group of the British Association for Sexual Health and HIV (BASHH) and the British HIV Association (BHIVA); 2010. January.

21. Ross J, Brady M, Clutterbuck D, Doyle T, Hart G, Hughes G, et al. BASHH recommendations for testing for sexually transmitted infections in men who have sex with men. British Association of Sexual Health and HIV; 2014.

22. Frankis J, Goodall L, Clutterbuck D, Abubakari R, Flowers P. Regular STI testing amongst men who have sex with men and use social media is suboptimal – a cross-sectional study. International Journal of STD & AIDS. 2017;28(6):573–583.

23. Harrison WO, Hooper RR, Wiesner PJ, Campbell AF, Karney WW, Reynolds GH, et al. A trial of minocycline given after exposure to prevent gonorrhea. New England Journal of Medicine. 1979;300(19):1074–1078.

24. McCutchan JA. Epidemiology of venereal urethritis: comparison of gonorrhea and nongonococcal urethritis. Reviews of Infectious Diseases. 1984;6(5):669–688.

25. Korenromp EL, Sudaryo MK, de Vlas SJ, Gray RH, Sewankambo NK, Serwadda D, et al. What proportion of episodes of gonorrhoea and chlamydia becomes symptomatic? International Journal of STD and AIDS. 2002;13(2):91–101.

26. Fifer H, Saunders J, Soni S, Sadiq ST, FitzGerald M. 2018 UK national guideline for the management of infection with Neisseria gonorrhoeae. International Journal of STD & AIDS. 2020;31(1):4–15.
